# The Principles of Cancer and Tumour Formation

**Published:** 1888-09

**Authors:** 


*The Principles of Cancer and Tumour Formation.* By W. ROGER WILLIAMS, F.R.C.S. Pp. 194. London: JOHN BALE & SONS. 1888.

"The present work" (the author tells us) "is intended as an introduction to a contemplated treatise on the Pathology and Treatment of Cancer and Tumour Formation, of which it forms the first part. The author proposes to complete the treatise in six parts." It is not easy to form an opinion of an edifice before the first story has been built; and it is not unlikely that if Mr. Williams had ventured to give us the whole of his work in one volume (it need not have been a large one, judging from the size of the first sixth part), we should have been more pleased with it. But Mr. Williams evidently likes to publish in driblets, as witness a somewhat imposing array of "Other Publications by the Author" on the last page, few of which could fill half-a-dozen pages octavo. Still, pregnant truths may be conveyed in few words.

The work before us contains a good deal of solid pabulum, served up with some sauce of speculation, and not a little spice of philosophy. For example of the last: "The *vis inertiæ* opposed to all progress is immense." "Empiricism is itself but a stepping-stone to real science. Those who think that the mere heaping up of facts constitutes science are certainly in error. We ought to remember what Aristotle long ago pointed out, that intellect (*νοῦς*) is the beginning and end of science." Such remarks—and the book contains many of them—we have found diverting and instructive, considerably adding to the zest of perusal. If they are seldom new, they are nearly always apt and to the point; a sort of Uncle Esek's Wisdom interspersed in a treatise on political economy.

The proper substance of the book is worked out with care and ability. The illustrative facts are numerous, have been gathered from very wide sources, and are marshalled in clear, coherent, and consecutive manner. Occasionally the conclusions seem to overleap the facts, and the deductions to outrun the premises; but the whole is a suggestive piece of reading, well written, clearly expressed, and exhibiting some logical acumen.

